# Exposure to bile and gastric juice can impact the aerodigestive microbiome in people with cystic fibrosis

**DOI:** 10.1038/s41598-022-15375-4

**Published:** 2022-06-30

**Authors:** Hafez Al-Momani, Audrey Perry, Andrew Nelson, Christopher J. Stewart, Rhys Jones, Amaran Krishnan, Andrew Robertson, Stephen Bourke, Simon Doe, Stephen Cummings, Alan Anderson, Tara Forrest, Ian Forrest, Michael Griffin, Matthew Wilcox, Malcolm Brodlie, Jeffrey Pearson, Christopher Ward

**Affiliations:** 1 Institutes of Cellular Medicine and Cell & Molecular Biosciences, Newcastle University Medical School, Newcastle University, Newcastle upon Tyne, NE2 4HH UK; 2 Department of Microbiology, Freeman Hospital, Newcastle upon Tyne Hospitals NHS Foundation Trust, Newcastle upon Tyne, NE7 7DN UK; 3 Faculty of Health and Life Sciences, Northumbria University, Ellison Place, Newcastle upon Tyne, NE1 8ST UK; 4 Adult Cystic Fibrosis Centre and Northern Oesophago-Gastric Unit, Royal Victoria Infirmary, Newcastle upon Tyne, NE1 4LP UK; 5 School of Science and Engineering, Teesside University, Middlesbrough, TS1 3BA UK; 6 Basic Medical Science Department, School of Medicine, The Hashemite University, Zarqa, Jordan; 7 Paediatric Respiratory Medicine, Great North Children’s Hospital, Newcastle upon Tyne Hospitals, Newcastle upon Tyne, UK; 8 NHS Fife and Newcastle University Medical School, Newcastle University, Newcastle upon Tyne, NE2 4HH UK; 9 Translational and Clinical Research Institute, Newcastle University, Newcastle upon Tyne, NE2 4HH UK

**Keywords:** Cell biology, Microbiology, Physiology, Medical research

## Abstract

Studies of microbiota reveal inter-relationships between the microbiomes of the gut and lungs. This relationship may influence the progression of lung disease, particularly in patients with cystic fibrosis (CF), who often experience extraoesophageal reflux (EOR). Despite identifying this relationship, it is not well characterised. Our hypothesis is that the gastric and lung microbiomes in CF are related, with the potential for aerodigestive pathophysiology. We evaluated gastric and sputum bacterial communities by culture and 16S rRNA gene sequencing in 13 CF patients. Impacts of varying levels of bile acids, pepsin and pH on patient isolates of *Pseudomonas aeruginosa* (Pa) were evaluated. Clonally related strains of Pa and NTM were identified in gastric and sputum samples from patients with symptoms of EOR. Bacterial diversity was more pronounced in sputa compared to gastric juice. Gastric and lung bile and pepsin levels were associated with Pa biofilm formation. Analysis of the aerodigestive microbiomes of CF patients with negative sputa indicates that the gut can be a reservoir of Pa and NTM. This combined with the CF patient’s symptoms of reflux and potential aspiration, highlights the possibility of communication between microorganisms of the gut and the lungs. This phenomenon merits further research.

## Introduction

Cystic fibrosis (CF) is the most common genetic condition affecting Caucasians^1^. Patients treated at specialist CF facilities show better survival rates, with effective care for lung disease a primary goal^2^. The advantages offered by specialist CF facilities are also linked to care across multiple disciplines, with significance attributed to treatment of gastro-intestinal issues. This includes gastro-oesophageal reflux (GOR), which occurs in around 50% of people with CF^3^. A number of causes for GOR in CF are suggested. These include a greater transient relaxation of the lower oesophageal sphincter (LOS), lower LOS pressure and delayed stomach emptying. It is also possible that physiotherapeutic interventions and coughing create a higher gradient of pressure in the abdomino-thoracic region^4^, leading to reflux. The functional importance of this includes the finding that people with CF who experience GOR tend to have poorer lung function^5^.

Microaspiration and potential gut to lung microbial movement has been shown in radiolabel studies of normal subjects^6^ and it is suggested that people with CF suffer a high burden of aspiration linked with reflux^3^. The stomach is sometimes considered a hostile environment which does not allow microbial pathogen survival. In fact however microbes can flexibly withstand extreme conditions, surviving high pH levels, digestive enzymes, strong detergent, and host immune reactions^7^.

We have previously described clonally related, CF relevant microbiology in gastric juice and sputum samples from people with CF, including *Pseudomonas aeruginosa* (Pa), and nontuberculosis mycobacteria (NTM) among other genera^8,9^. GOR is a major co-morbidity in CF and is associated with the presence of bile acids in the lungs most likely by micro-aspiration. The repeatability of our recently described “CF aerodigestive microbiome” has not been documented and there is limited understanding of how the GI environment interacts with microbiological isolates obtained from CF patients. Bile acids (BAs) have been observed within sputum, bronchoalveolar lavage (BAL) and end stage lung disease in people with CF, at concentration levels between 0.4 μM^10^ and 1 mM^11–13^. In vitro studies have found that bovine bile can lead Pa and a number of pathogenic species to take up biofilm modality^14^.

Therefore, it is important to further explore how the lung and GI environment might interact with microbiological survival and biofilm growth of patient microorganisms, which are known to be associated with morbidity and mortality in CF lung disease. The aim of this paper therefore is to determine whether our finding of gastric and lung microbiomes were repeatable in people with CF. Complimentary laboratory experimental studies on Pa isolates from CF patients were also performed to investigate Pa lifestyle in simulated aerodigestive environments and potential formation of biofilms.

## Materials and methods

### Patient cohorts

This study was approved by the Newcastle University Biobank and the Newcastle and North Tyneside 1 Research Ethics Committee. All study participants provided written informed consent prior to initiation of the study. All methods were carried out in accordance with relevant guidelines and regulations.

CF patients receiving percutaneous endoscopic gastrostomy (PEG) feeding represented an important opportunity to directly sample gastric juice, with less potential for contamination with oropharnygeal commensals. From the 270 CF patients attending the regional CF Centre, all adult stable CF patients receiving PEG tube feeding, were therefore included in this study; 13 patients who attended the clinic during the course of the study between 22/05/2013 and 17-04/2014 were studied. No PEG fed CF patients were excluded or positively selected for the study.

### Patient samples and molecular based studies

Two sets of gastric juice and sputum samples were collected 6 months apart, (T1 and T2) from all (n = 13) available CF patients fed via percutaneous endoscopic gastrostomy (PEG) tube in our regional CF centre. Methods previously outlined^9^ were used to analyse gastric juice and sputum microbes. Bacterial profiling of the variable region 4 (V4) of the 16S rRNA gene was carried out by NU-OMICS (Northumbria University) based on the Schloss wet-lab MiSeq SOP^15^. Briefly, PCR was carried out using 1× Accuprime Pfx Supermix, 0.5 µM each primer and 1 µl of template DNA under the following conditions 95 °C 2 min, 30 cycles 95 °C 20 s, 55 °C 15 s, 72 °C 5 min with a final extension 72 °C 10 min. One positive and one negative control sample were included in each 96 well plate and carried through to sequencing. PCR products were normalised using SequalPrep™ Normalization kit (Invitrogen) as described in the manufacturer’s instructions and combined into four pools. Pools were combined in equimolar amounts to create a single library then denatured using 0.2 N NaOH for 5 min followed by a 2 min incubation at 96 °C. The library was diluted to a final concentration of 4.5 pM, supplemented with 5% PhiX and loaded onto a MiSeq V2 500 cycle cartridge, Each pool was quantified using fragment size determined by BioAnalyzer (Agilent Technologies) and concentration by Kappa qPCR (Kappa Biosystems).

Raw fastq files were processed using Mothur (v1.35.1) as described in the MiSeq SOP^15^. Sequences were aligned to the Silva database and chimeric sequences were detected by Chimera.uchime and removed from downstream analysis. OTUs were selected at a 97% similarity threshold and taxonomy was assigned using the Ribosomal database project 16S rRNA reference (v16) using a cut off value of 70 producing a total of 4,366,517 reads. The samples were subsampled using the sub.sample command in Mothur to 1000 reads per sample.

### Pa and NTM strain typing

Strain typing was performed using methods previously outlined (13). All isolates were identified by matrix assisted laser desorption ionisation time-of-flight (MALDI-TOF) mass spectrometry (Bruker Daltonics, UK). *Pa* and mycobacteria were strain typed using variable number tandem repeat (VNTR) analysis, (Colindale, UK), independently of the author’s laboratories. For PA: amplification of 9 VNTR loci (ms61, ms172, ms207, ms209, ms211, ms213, ms214, ms217 and ms222)as described by Vu-Thien et al.^16^ and Onteniente et al.^17^. Mycobacterium was identified by *rpo*B, *sodA* and *hsp*65 gene sequencing and strain typed using amplification of 9 VNTR loci (3416, 4356, 3163, 4038, 4093, 3320,2177,3398, 2220) for variable number repeat analysis (VNTR), Colindale, UK^18,19^.

### Whole genome sequencing (WGS)

In 2 patients, where clonally related Pa was identified in gastric juice and lung samples, WGS was used to confirm the close relationship between the isolates. In brief DNA was extracted from selected isolates using the QIAGEN PowerLyzer Power soil kit. Libraries were prepared from 1 ng of extracted DNA using the Nextera XT kit (Illumina) and sequenced by NU-OMICs using the V2 500 cycle reagent kit on the MiSeq benchtop sequencer.

**Figure 1:**
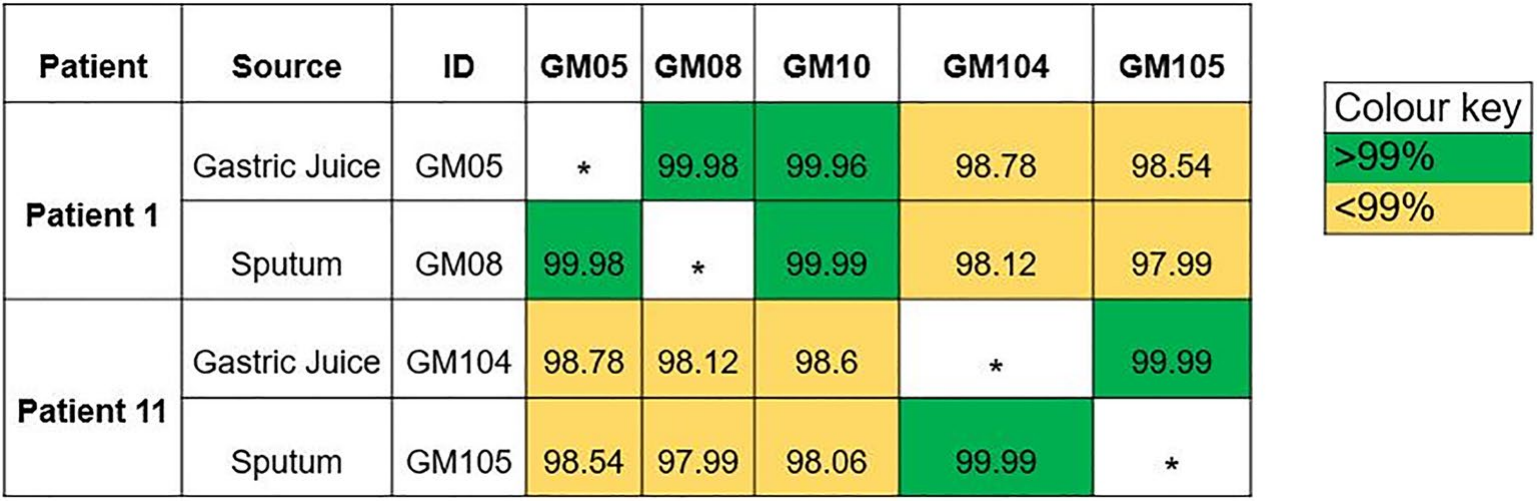
Average Nucleotide Identity pairwise comparisons of *Pseudomonas aeruginosa* isolate genomes from patient 1 and patient 11 lung and gastric juice samples. Comparisons coloured green have > 99% similarity and comparisons coloured yellow have < 99% similarity.

The forward and reverse reads were checked for quality using FastQC and trimmed using Trimmomatic (v0.38.1) to remove any remaining Nextera adapter sequences and a sliding window of Q20 across 4 bp. The trimmed sequences were used for assembly using SPAdes (v3.12.0) with careful correction enabled and kmer detection of 21, 33, 55 and contigs less than 1000 bp were removed. The quality of assemblies was assessed using QUAST (v4.4) and CheckM (v1.0.18) (Supplementary Table S1). The assembles were corrected in Mauve using the Move contigs algorithm with Pseudomonas aeruginosa C-NN2 (Accession NZ_LT883143) identified as an appropriate reference from VNTR typing. The Average Nucleotide Identity (ANI) between draft genomes from each of the isolates from the same patient were generated using ANI Calculator with a minimum contig length of 1000 bp^20,21^ and window size of 1000 bp and a step size of 200 bp (Fig. 1).

The raw data for the 16S rRNA gene V4 amplicon sequencing and the genome assemblies is deposited to the NCBI BioSample database with accession numbers SAMN26565353–SAMN26565426 (https://www.ncbi.nlm.nih.gov/biosample/).

### Symptoms of extra oesophageal reflux

Patient symptoms of extraoesophageal reflux (EOR) were assessed using the Reflux Symptoms Index (RSI) score. The RSI is a simple validated^22^, questionnaire consisting of nine questions regarding the symptoms of laryngopharyngeal reflux disease (LPRD), where the patient is asked to score from zero to five depending on the severity of the symptom. An RSI of > 13 is considered as abnormal^22^ (Supplementary Appendix 1).

### Experimental evaluation of *P. aeruginosa* lifestyle in models of the human aerodigestive environment

#### Materials and chemicals

All reagents were obtained from Sigma-Aldrich, Poole, Dorset, UK. To reach pH targets ranging from 2.5 to 7.4, 1 Molar HCl was added to Phosphate-buffered saline (PBS), and this was then used to create porcine pepsin solutions of between 0.5 and 1.5 mg/ml. Four bile acids; lithocholic acid (LCA), cholic acid (CA), deoxycholic acid (DCA) and chenodeoxycholic acid (CDCA) were used ; from 0.3 to 20 mM/l, in line with gastric concentrations^23,24^, and from 9.4 to 150 µM/l, for lung informed by BAL measurements in people with CF^10–13^.

### Strains of bacteria and cultures used

Pa strains Pa01, S33, S34 and Pa14 were used. S33 and S34 were isolated from 2 CF gastric juice samples^9^. Isolates reference Pa01 and Pa14 are from the International panel of Pa^25^. Columbia blood agar was used for subculturing the strains at 37 °C in the presence of oxygen until the next day.

### Establishing effects of pepsin and acidity level on *P. aeruginosa*

A 10 µl Pa Inoculum was taken from a 0.5 McFarland standard Pa suspension (comparable density suspension 1.5 × 10^8^ cfu/ml), pre-incubated with a 1 ml solution containing the test challenge substances, with 10 µl being taken at durations of 0 min, 5, 30, 60 and 120 min. These underwent dilution in 1.98 ml PBS, pH 7.4. 50 µl of the neutralised suspension of bacteria was taken for plating in blood agar, and this was done in triplicate, to give about 50–60 Pa colonies. Viable counts were determined after culturing at 37 °C for 24 h under aerobic conditions (Supplementary Appendix 2A).

### Biofilm assay: microtiter-plate test

The biofilm assay used a standard microtiter plate assay using a crystal violet (cv) based staining protocol quantified by changes in optical density (Supplementary Appendix 2B). The cv staining method, originally described by O’Toole and Kolter in 1998 to identify biofilm-deficient mutants^26^, has become the clinical microbiology lab “gold standard” for quantifying biofilms in a microtitre dish. It is an inexpensive assay that can be routinely performed with relative ease^27^, can be used for both Gram-positive and Gram-negative organisms, and is suitable for qualitative and quantitative measurements of bacterial cells adherence to a variety of surfaces^28^. In spite of its popularity, the CV technique is an indirect measure of biofilm formation as it can measure adherence of bacterial cells that adhere to surfaces without forming biofilms^29^. These issues may contribute to a large variability between samples that may complicate the interpretation of biofilm screening results.

### Statistical analysis

Bland and Altman plots were used to assess agreement between Shannon diversity measures at T1 and T2 of repeated samples. This methodology has been widely used to assesses agreement between repeated biological measures^30,31^.

Data were summarised graphically and statistically analysed via analysis of variance (ANOVA). The Siegal–Tukey test was employed for comparison of OD measurements from microtiter plate testing for samples containing bile and those which did not. The t-test was used for post hoc two-by-two comparisons to compliment the ANOVA approach. Statistical significance was assumed for all findings with a p value equal to or smaller than 0.05.

## Results

### CF patients and symptoms of extra oesophageal reflux (EOR)

The patients in this investigation, with a median age of 25 (range 18–32 year) demonstrated moderate to severe CF lung disease, common amongst the PEG-fed CF population (median forced expiratory volume in one second (FEV_1_) at T1, 1.65 l (38% predicted (compared to normal based on height, weight, and race) range 0.5–3.5 l (12–88%); median FEV_1_ at T2, 1.35 l (42% predicted) range 0.46–3.25 l (11–82%). They also showed low body mass index (BMI) (median at T1 19.4, range 15.2–23.2; median at T2 19.3, range 15.3–22.8). All were long term users of antibiotics (average use 70 days/year, range 14–197 at T1, and 79 days/year, range 14–190 at T2). All used acid suppressants and were EOR symptomatic; median RSI score 17 (range 13–36, n = 13). Table 1 contains the clinical details of the CF patients included in this study.

**Table 1:** Demographic and clinical data for CF patients at T1 and at T2.

Patient	Genetics	Age	RSI score	PPI yes/no	GJ pH	FEV1 (% pred) at T1	FEV1 (% pred) at T2	BMI at T1	BMI at T2	IV days /year	Long-term antibiotic
CF-1	F508del/F508del	26	17	Yes	6	2.0 L (52%)	1.61 (42%)	19.9	18.7	22	Azith Inh Coli Inh Tob
CF-2	F508del/F508del	27	20	Yes	2	1.7 L (42%)	1.67 (42%)	23.2	22.8	70	Azith Fluclox Inh coli
CF-3	F508del/F508del	20	25	Ranitidin	3	0.8 L (26%)	0.7 (22%)	19.5	20	28	Azith Fluclox Inh Coli
CF-4	F508del/F508del	24	36	Yes	6	0.76 L (28%)	0.65 (23%)	19	19.3	154	Azith Inh Coli
CF-5	F508del/F508del	31	16	Yes	6	0.5 L (12%)	0.46 (11%)	19.1	19.3	70	Azith Inh Coli
CF-6	F508del/R117H	22	16	Yes	3	2.7 L (66%)	2.6 (63%)	16.4	16.5	14	Fluclox
CF-7	I507del/Arg560Lys	18	13	Yes	2	3.5 L (88%)	3.25 (82%)	19.4	20.2	37	Fluclox Inh Coli Inh Tob
CF-8	F508del/R117H	30	14	Yes	6	1.55 L (46%)	1.4 (42%)	17.8	17.7	56	Fluclox Inh Coli
CF-9	F508del/F508del	25	17	Yes	2	1.7 L (38%)	1.25 (25%)	15.9	16.3	98	Azith Fluclox Inh Tob
CF-10	F508del/G542X	32	NA	Yes	2	1.15 L (36%)	1.35 (42%)	19.4	20	112	Azith Inh Coli
CF-11	F508del/F508del	30	19	Yes	6	1.2 L (29%)	1.1 (27%)	19.8	19.8	115	Azith Fluclox Inh Coli
CF-12	F508del/G542X	24	15	Yes	2	1.65 (36%)	0.9 (20%)	15.24	15.3	197	Azith Inh Coli Inh Tob
CF-13	F508del/Arg851Ter	23	22	Yes	6	2.3 (59%)	2.1 (52%)	20.2	20	56	Azith

*FEV1* forced expiratory volume in 1 s, *Azith* oral azithromycin long-term, *Fluclox* oral flucloxacillin long-term, *Inh Coli* inhaled colistin (nebulised or inhaler), *Inh Tob* inhaled tobramycin (nebulised or inhaler).

### Comparing Pa isolated from gastric juice and sputum samples

Gastric juice and sputum samples from 13 CF patients (CF1–13) were taken on two separate occasions (T1 and T2) 6 months apart.

Pa were isolated from 2 out of 13 gastric juice samples (CF1 and CF11) at T1 and in 2 different patients at T2 (CF5 and CF8). In CF sputum samples, Pa were isolated from 9 CF patients at T1 (CF1, 5, 7–13) and from 5 CF patients at T2 (CF3, 8, 9, 11, 12).

Comparing the paired bacterial profile isolated from the gastric juice and sputum sample from the same patient showed that Pa were clonally related (confirmed by variable number tandem repeat (VNTR) between gastric juice and sputum samples in 2 patients (CF1 and 11) at T1 and in 1 patient at T2 (CF8) (Tables 2, 3).

**Table 2:** *Pa* and NTM status among CF gastric juice (GJ) and sputum samples taken at different time points 6 months apart (T1 and T2) using routine culture approaches previously outlined^9^.

	Baseline NTM status	Baseline Pa status	Sputum time 1	Gastric juice T1	Sputum time 2	Gastric juice T2
CF1	No previous	Chronic related	Pa	Pa	− ve	− ve
CF2	No previous	No previous	− ve	− ve	− ve	− ve
CF3	Chronic related	Chronic related	− ve	− ve	Pa *Mycobacteroides massiliense*	*Mycobacteroides massiliense*
CF4	Chronic related	No previous	*M. abscessus *subsp.* abscessus*	− ve	*M. abscessus *subsp.* abscessus*	− ve
CF5	No previous	No previous	Pa	− ve	− ve	Pa
CF6	Chronic related	No previous	*M. abscessus *subsp.* abscessus*	− ve	− ve	− ve
CF7	No previous	Chronic related	Pa	− ve	N/A	N/A
CF8	No previous	Chronic related	Pa	− ve	Pa	Pa
CF9	No previous	Chronic related	Pa	− ve	Pa *Mycobacteroides massiliense*	− ve
CF10	No previous	Chronic related	Pa	− ve	N/A	N/A
CF11	No previous	Chronic related	Pa	Pa	Pa	− ve
CF12	No previous	Chronic related	Pa	− ve	Pa	− ve
CF13	No previous	Chronic related	Pa	− ve	*Mycobacteroides massiliense*	*Mycobacteroides massiliense*

Cultures which were negative for Pa and NTM are denoted as –ve, N/A indicates that culture data is unavailable. Chronic related indicates where the organisms were present over time, and shown to be related to previous isolates from the same patient (VNTR).

**Table 3:** VNTR profile for PA isolated form Gastric juice and sputum sample of patient 1 and 11.

Patient	Source	Species	Number of copies VNTR region								
			127	211	213	214	217	222	207	209	61
CF1	Gastric juice	PA	9	2	3	–	3	2	5	–	10
	Sputum	PA	9	2	3	–	3	2	5	–	10
CF11	Gastric juice	PA	11	–	–	2	1	3	7	3	12
	sputum	PA	11	–	–	2	1	3	7	3	12

### Whole genome sequencing

In two patients where clonally related Pa were identified in gastric juice and lung samples by VNTR (patients 1 and 11, T1) (Table 3). WGS was used to confirm this close relationship between the isolates. The mean ANI for these isolates was 99.99% for patient 1 and 99.98% for patient 11. Figure 1 shows a representative illustration of the WGS data (patient 11, T1) showing a close relationship between the annotated genomes.

### Comparing NTM in repeated gastric juice and sputum samples

Gastric juice and sputum samples from 13 CF patients (CF1–13) were taken on two separate occasions (T1 and T2) 6 months apart and compared for the presence of NTM. Cross sectional data at T2 have been previously published^8^.

Comparing the microbial profiles isolated from the gastric juice and sputum sample from the same patients showed that NTM were isolated among 5 different patients (Table 2).

At T1, only 2 patients had *Mycobacteroides abscessus* subsp. *abscessus* in their sputum (CF4 and 6), while at T2, a higher prevalence of NTM was detected compared to T1 with four out of 13 CF patients (four in their sputum and 2 of them had NTM in their gastric juice) having detectable NTM (CF 3, 4, 9, and 13).

Among those patients with sputum colonisation NTM at T2, 4 patients had NTM colonisation in their sputum, 3 patients with *Mycobacteroides abscessus* subsp. *massiliense* (CF3, CF9, CF13) and 1 with *Mycobacteroides abscessus* subsp. *abscessus* (CF4). Among the patients with sputum isolates, 2 had the same strain of NTM confirmed by VNTR in their gastric juice (*M. massiliense* in CF3 and CF13) (Tables 2, 4).

**Table 4:** VNTR profile for NTM species isolated form gastric juice and sputum sample of patient 3 and 13.

Patient	Source	NTM species	Number of copies VNTR region								
			3416	4356	3163	4038	4093	3320	2177	3398	2220
CF3	Gastric juice	*Mycobacterium massiliense*	1+	–	2	2	1	2	1+	1	2
	Sputum	*Mycobacterium massiliense*	1+	–	2	2	1	2	1+	1	2
CF13	Gastric juice	*Mycobacterium massiliense*	1+	–	1+	3	2	3	2+	2+	–
	sputum	*Mycobacterium massiliense*	1+	–	1+	3	2	3	2+	2+	–

### Bacterial profiles of repeated gastric juice and sputum microbiome samples based on 16S rRNA gene sequencing

**Figure 2:**
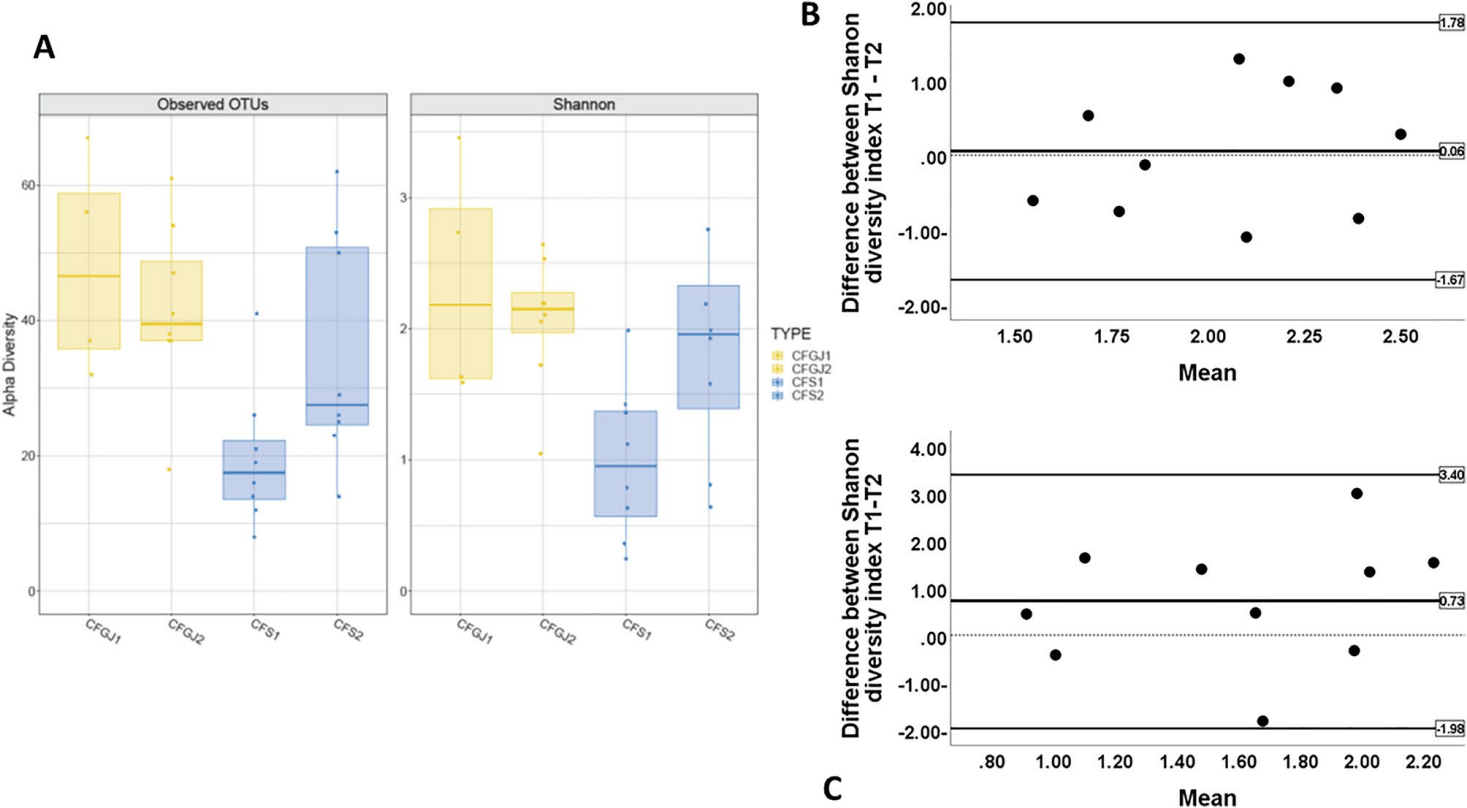
(**A**) Alpha diversity (left) and Shannon diversity index (right) of repeated CF gastric juice (CFGJ) and CF sputum (CFS) samples at the two time points (T1 and T2, n = 10). (**B**) Bland Altman plot of the Shannon diversity index of gastric juice samples (n = 10) from CF patients at 2 time points (T1 and T2). The mean of the Shannon diversity of each subject (x-axis) is plotted against the differences between T1 and T2 (y-axis). Mean difference between T1 and T2 = 0.14 (SD 0.86). (**C**) Bland Altman plot of the Shannon diversity index sputum samples (n = 10) from CF patients at 2 time points (T1 and T2). The mean of the Shannon diversity of each subject (x-axis) is plotted against the differences between T1 and T2 (y-axis). Mean difference between T1 and T2 = 0.72 (SD 1.35).

There was insufficient sample for 3 patients. Ten paired sputa and gastric juice samples were therefore available for molecular analysis. The two patient sampling time points measured by alpha diversity and Shannon diversity index, showed biological variability. The CF sputum tended to have higher but not statistically significant microbial diversity at T2 (Fig. 2A). In comparison with sputum samples, the gastric juice samples showed less variation in alpha diversity between time points (Fig. 2A, Supplementary Fig. S1), Bland and Altman plots indicated greater fluctuations in the Shannon diversity index in sputum samples (Figs. 2C) compared to gastric juice samples (Figs. 2B, Supplementary Fig. S1). PERMANOVA analyses were used to compare bacterial profiles in CF gastric juice and sputa (Supplementary Fig. S2), this indicated that the centroids and spread of the profiles overlapped between the groups (p = 0.08).

### Pa lifestyle in simulated aerodigestive environments

Experimental work investigating tolerance of acidity for the 4 strains of Pa in PBS and using pH conditions of 7.4 as a control with which to compare the results, show that below pH 4, a significant growth inhibition of Pa was achieved at 2 h (F (6,14) = 4.866, p < 0.001). This significant effect also appeared at lower pH values, with shorter incubation times. (Supplementary Fig. S3).

### Effects of pepsin

Incubating Pa in PBS containing porcine pepsin at 0.5, 1 and 1.5 mg/ml, at a range of pH levels demonstrated that all 4 Pa strains were sensitive to proteolysis. At a pH level of 3, the samples displayed a large decrease in viable microbes (colony number), and the majority had not survived a 15 min incubation (Supplementary Fig. S4). There was also a complete loss of bacteria where pH was 3.5, and at 1 and 1.5 mg of porcine pepsin, all had been killed by 60 min’ incubation, whereas for 0.5 mg, this had been achieved by 120 min (Supplementary Fig. S5).

For samples incubated at pH 4, porcine pepsin had a lesser impact on Pa strains following 15-min. Approximately 15% of bacterial cells were killed at 15 min where pepsin was present, and 30% at 120 min. In the non-pepsin sample, just 12% were killed (Supplementary Fig. S6). There was no significant effect of pepsin on bacteria at pH 5 (F (3,16) = [1.906], p = 0.169) (Supplementary Fig. S7).

### The impact of bile acids at concentrations found in gastric juice on Pa growth

**Figure 3:**
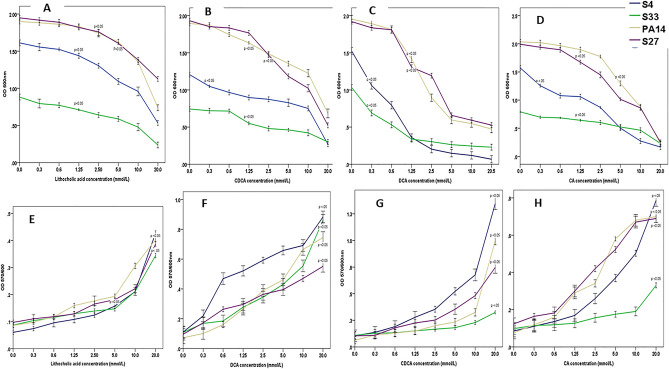
The Impact of different bile acids at concentrations range 0.3 mmol/l to 20mmmol/l (x-axis) (resembling the concentration of bile acid at gastric juice in normal healthy individual on *Pa* growth (**A–D**) and biofilm formation (**E–H**) (*OD* optical density).

Incubation of Pa with four different bile acids in concentrations from 0.3 to 20 mmol/l had a negative impact on growth, although there was variability between strains. Lithocholic acid reduced growth significantly (p < 0.05) in two of the strains of Pa (S33 and S34) at a 1.25 mmol/l concentration or above, while the remaining two strains were impacted when the concentration reached 5 mmol/l or above (F ((4, 16.64) = [6.659], (p = 0.002) (Fig. 3A). Incubation with DCA reduced growth significantly for two of the strains (S33 and S34), at the lowest concentration of 0.3 mmol/l, while for the remaining two strains, growth was reduced significantly (F (2,93) = [39.86], (p = 0.005) when DCA concentrations were over 1.25 mmol/l (Fig. 3B). Both CDCA (Fig. 3C) and CA (Fig. 3D) concentrations of over 2.5 mmol/l (F (7, 21) = [16.33], p = 0.009) and 5 mmol/l significantly (F (7,24) = [3.745], (p = 0.007) reduced the growth of Pa, respectively. There was therefore variability among different strains in term of bile tolerance.

The Impact of bile acids at concentrations found in gastric juice indicated increased formation of Pa biofilm, for each strain, at varying bile acids concentrations (Fig. 3E–H; increase in OD 570/600 values). Statistically significant increases in biofilm formation were observed at 20 mmol/l for all bile acids (F(2,18) = 6.576, p = 0.033) (Fig. 3E–H).

### Impact of bile acids, at concentrations found in lung, on Pa growth and biofilm formation

**Figure 4:**
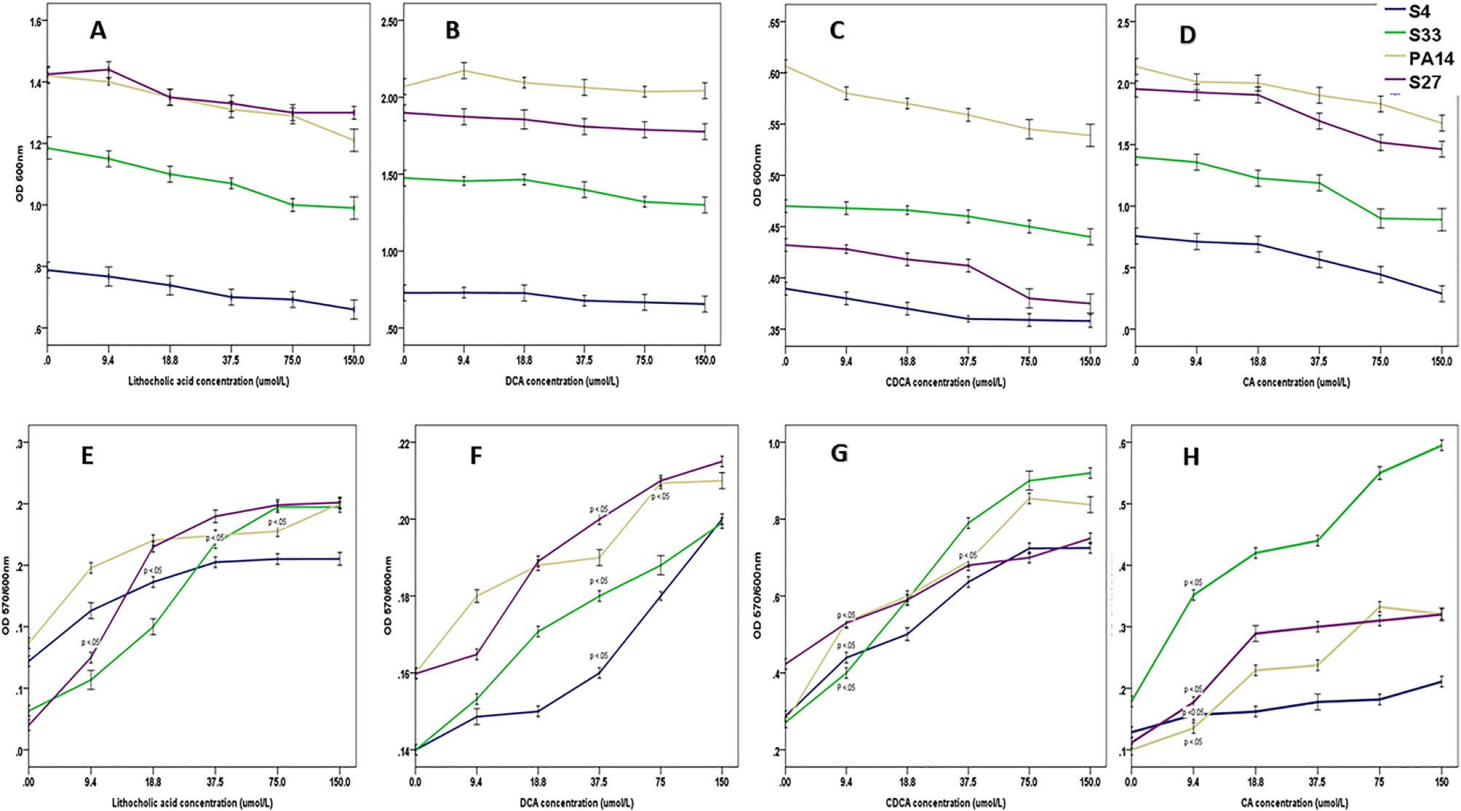
The impact of different bile acids on *Pa* growth (**A–D**) and biofilm formation (**E–H**), at concentrations detected in sputum and bronchoalveolar lavage (BAL) in CF.

When Pa was incubated with bile acid levels at CF lung relevant levels informed by BAL (9.4 µmol/l to 150 µmol/l), Pa growth was not altered (Fig. 4A–D). There was a significant increase (F (4,9.5) = [9.951], p = 0.007) in biofilm formation however (Fig. 4E–H), with variability in the strain response to bile acids. 9.951 (4.000, 9.477).

## Discussion

Our study provides novel data repeatedly characterising gastric and lung microbiomes in people with CF. Pathogens including clonally related strains of Pa and NTM were isolated in gastric and lung samples from patients with symptoms of EOR, with variation over time. Exposure to bile and pepsin levels relevant to gastric and lung compartments were associated with Pa biofilm formation. It is likely that gastric juice Pa and NTM may derive from swallowed sputum containing *Pa* that is cleared from the lung following cough. However, We previously found that the digestive tract and airways were populated by bacteria which were sometimes related and of known importance in CF lung disease, including biofilm forming strains of *Pa*^32,33^. This highlights the possibility that the stomach could constitute a viable bacterial reservoir, relevant to the overall pathophysiology of CF. Our data indicate that although Gastric juice *Pa* may be swallowed following mucocilliary clearance of a diseased airway, *Pa* could also be derived through diverse routes and sources, including but not restricted to microaspiration for lung *Pa*. These findings may be relevant for detection, surveillance and eradication of organisms and suggests potential gut to lung transmission following aspiration, which warrants further study.

A relationship between aspiration of gastric contents during a reflux event and deterioration of lung function is implied by the finding of poorer lung function in CF patients with acid reflux^5^. Our study confirmed a high burden of gastrointestinal symptoms in people with CF^34^ and suggests that the connection between the airways and the stomach may be relevant to lung infection^9,35^. We propose that cough and other symptoms of EOR mean that a gastric reservoir could be relevant to transfer of organisms within and between patients. Cough aerosols, fomite transmission and EOR and aspiration represent potential mechanisms of transfer that could be investigated in future research.

There is increasing awareness that both the gut and lung microbiome may have an important role in overall CF pathophysiology, but there are limited numbers of studies^36^. In a previous paper from our group clonally related strains of Pa were identified in sputum and gastric juice in 4 people with CF^9^. Our present longitudinal follow up study confirmed that a closely related Pa was present in the sputum and gastric juice of some patients. Moreover, two patients had clonally related strains of *M. massiliense* in their sputum and gastric juice. Our present study builds on and strengthens our previous findings, using whole genome sequencing to confirm that Pa found in both gut and lung were closely related, confirming the VNTR results with a different analytical platform and analysis.

The swallowing of expectorated sputa is a normal homeostatic mechanism that could lead to the isolation of clonally related microorganisms in the gut and lung environments. Previous studies have also indicated the possibility of microflora exchange between the stomach and the lungs, in children with chronic cough^37^. It is of interest that previous radioisotope studies have shown that 50% of adult normal volunteers had gastric tracer in the lung following sleep, with the quantities of tracer aspirated ranging from 0.01 to 0.2 ml^38^. This order of magnitude was discussed as “likely to contain bacterial organisms in physiologically significant quantities”^39^. In our study Identification of patients who had gastric samples with NTM but who had negative sputa, indicate the possibility that NTM gastric isolates may be independent from respiratory sources.

Our description of an aerodigestive microbiome prompted us to perform complimentary laboratory based work that investigated the potential for lung and gastric environments to interact with microbes. We demonstrated a significant bactericidal impact for Pa in acidic conditions < pH 3 but at higher pH levels, the impact was small. When pepsin is present in acid conditions, Pa were more effectively destroyed. Other research into the impact of acidity on bacteria in *Serratia marcescens* are consistent with our findings^40^.

Thus, the effectiveness of the stomach’s bacterial barrier function varies with pH. The pH of the stomach can be modulated physiologically and with medication. Food entering the stomach, can contain bacteria and lower acidity, bringing pH to between 3 and 4.5. Pathogens which are bound to components of the food in the stomach find some protection from acidity in this way^41^. Pepsin concentration is a second element in bactericidal activity in the stomach, with effectiveness much more notable at levels > 1.0 mg/ml compared with 0.5 mg/ml in our data.

Gastric pH can also be altered by therapy. According to the United States CF Foundation Patient Registry, 70% of patients were treated with medication to block gastric acid, while Com et al. report up to 100% of patients are treated at some centres^42^. This is often for symptoms of GOR.

CF patients may also take acid inhibition medication to enhance the efficacy of pancreatic enzyme replacement therapy; a mainstay of modern CF patient care, associated with better life expectancy^43,44^. While generally considered a safe therapeutic approach, the role of ant-acid treatment and reflux in CF is an area of current debate^45,46^. Recent reviews and patient and public involvement identify this as an important research gap^47^.

We have previously accurately quantified the presence of lung bile acids in people with CF before and after lung transplant^11,13^. This prompted us to model the effect of bile acids on patient-derived organisms of the aerodigestive microbiome, complementing our work on pH and pepsin. At concentrations similar to those in the stomach, bile acids had a potential negative impact on Pa growth, with a tendency to form a biofilm. Interestingly there was variability between strains in this. Different, lab reference and clinically derived Pa strains were investigated, which seemed to have different responses to bile acids. This could be investigated further in future studies.

The behaviour of Pa was different when challenged with lower concentrations of bile acids, analogous to lung levels. Pa growth was unchanged, but a rise in biofilm formation was observed. The findings suggest the presence of bile acids within the lungs may be a significant environmental pressure on Pa phenotype. This could be a contributory factor to the decrease in lung function and morbidity observed in CF respiratory disease^14,48^. Our results support reports that a bovine bile concentration of 0.1–1 mmol/ml causes pathogenic microbes, including Pa, to move into a chronic infective^14^, biofilm form, with antibiotic resistance^49^. This process may be dependent on the production of pseudomonas quinolone signal (PQS), produced in the presence of bovine bile salts^14,50^.

We postulate that our findings may be particularly important if bacteria enter the lungs as a direct inoculum following gastric reflux and aspiration. It is possible that Pa in the stomach, converted to biofilm by the effect of high bile acid levels, is refluxed into the airways, where bile acids represent an ongoing environmental pressure to drive and maintain Pa biofilms. Pa entering the lung from sources other than the stomach may also be converted to biofilm by the effect of the low level of bile acid present in the airway environment.

Our study has limitations. Our descriptive, associative approach cannot prove gastric to lung transmission of organisms and although we collected samples from all available CF patients fed via PEG in our regional CF centre, we recognise this is a small number of patients. We made an informed choice to study PEG-fed patients to mitigate against the possibility of commensalism in our gastric microbiology. It could therefore be argued that our results may be limited to the PEG-fed population studied. Our study population had moderate to severe CF lung disease and may also have been at risk of NTM colonisation due to gastrostomy associated breach of barrier defence, which has been described in tuberculosis^51^.

The current study should stimulate further research into the mechanisms through which aspiration may lead to chronic inflammatory states and infections in CF. Further research into the relationship between the lung and stomach microbiomes could include extending the period of longitudinal follow up of CF patients. Patient derived isolates could also be grown within gastric fluid collected from people with CF, to determine how organisms may survive, through adaptation and biofilm formation. Such work could provide a novel route for developing new therapeutic interventions, which may also be relevant to other chronic lung diseases associated with reflux and aspiration^52^.

## Supplementary Information

Supplementary Information.

## References

[1] LiPuma JJ (2010). The changing microbial epidemiology in cystic fibrosis. Clin. Microbiol. Rev..

[2] Mahadeva R (1998). Clinical outcome in relation to care in centres specialising in cystic fibrosis: cross sectional studyCommentary: Management in paediatric and adult cystic fibrosis centres improves clinical outcome. BMJ.

[3] Blondeau K (2008). Gastro-oesophageal reflux and aspiration of gastric contents in adult patients with cystic fibrosis. Gut.

[4] Pauwels A (2011). Gastric emptying and different types of reflux in adult patients with cystic fibrosis. Aliment. Pharmacol. Ther..

[5] Navarro J (2001). Factors associated with poor pulmonary function: Cross-sectional analysis of data from the ERCF. Eur. Respir. J..

[6] Gleeson K, Maxwell SL, Eggli DF (1997). Quantitative aspiration during sleep in normal subjects. Chest.

[7] Sachs G, Scott D, Weeks D, Melchers K (2000). Gastric habitation by *Helicobacter pylori*: Insights into acid adaptation. J. Neurosci..

[8] Al-Momani H (2017). Nontuberculous mycobacteria in gastrostomy fed patients with cystic fibrosis. Sci. Rep..

[9] Al-Momani H (2016). Microbiological profiles of sputum and gastric juice aspirates in Cystic Fibrosis patients. Sci. Rep..

[10] Pauwels A (2012). Bile acids in sputum and increased airway inflammation in patients with cystic fibrosis. Chest J..

[11] Aseeri A (2012). Bile acids are present in the lower airways of people with cystic fibrosis. Am. J. Respir. Crit. Care Med..

[12] D’Ovidio F (2005). Bile acid aspiration and the development of bronchiolitis obliterans after lung transplantation. J. Thorac. Cardiovasc. Surg..

[13] Brodlie M (2015). Bile acid aspiration in people with cystic fibrosis before and after lung transplantation. Eur. Respir. J..

[14] Reen FJ, Woods DF, Mooij MJ, Adams C, O'Gara F (2012). Respiratory pathogens adopt a chronic lifestyle in response to bile. PLoS ONE.

[15] Kozich JJ, Westcott SL, Baxter NT, Highlander SK, Schloss PD (2013). Development of a dual-index sequencing strategy and curation pipeline for analyzing amplicon sequence data on the MiSeq Illumina sequencing platform. Appl. Environ. Microbiol..

[16] Vu-Thien H (2007). Multiple-locus variable-number tandem-repeat analysis for longitudinal survey of sources of *Pseudomonas aeruginosa* infection in cystic fibrosis patients. J. Clin. Microbiol..

[17] Onteniente L, Brisse S, Tassios PT, Vergnaud G (2003). Evaluation of the polymorphisms associated with tandem repeats for *Pseudomonas aeruginosa* strain typing. J. Clin. Microbiol..

[18] Harris KA (2012). Molecular fingerprinting of *Mycobacterium abscessus* strains in a cohort of paediatric Cystic Fibrosis patients. J. Clin. Microbiol..

[19] Turton JF (2010). Evaluation of a nine-locus variable-number tandem-repeat scheme for typing of *Pseudomonas aeruginosa*. Clin. Microbiol. Infect..

[20] Rodriguez-R. L. M. & Konstantinidis, K. T. *The Enveomics Collection: A Toolbox for Specialized Analyses of Microbial Genomes and Metagenomes. Report No. 2167–9843* (PeerJ Preprints, 2016).

[21] Goris J (2007). DNA–DNA hybridization values and their relationship to whole-genome sequence similarities. Int. J. Syst. Evol. Microbiol..

[22] Belafsky PC, Postma GN, Koufman JA (2002). Validity and reliability of the reflux symptom index (RSI). J. Voice.

[23] Pearson JP, Parikh S (2011). Review article: Nature and properties of gastro-oesophageal and extra-oesophageal refluxate. Aliment. Pharmacol. Ther..

[24] Ali MS, Parikh S, Chater P, Pearson JP (2013). Bile acids in laryngopharyngeal refluxate: Will they enhance or attenuate the action of pepsin?. Laryngoscope.

[25] De Soyza A (2013). Developing an international *Pseudomonas aeruginosa* reference panel. Microbiologyopen.

[26] O'Toole GA, Kolter R (1998). Flagellar and twitching motility are necessary for *Pseudomonas aeruginosa* biofilm development. Mol. Microbiol..

[27] Haney EF, Trimble MJ, Cheng JT, Vallé Q, Hancock RE (2018). Critical assessment of methods to quantify biofilm growth and evaluate antibiofilm activity of host defence peptides. Biomolecules.

[28] O'Toole GA (2011). Microtiter dish biofilm formation assay. JoVE.

[29] Roy R, Tiwari M, Donelli G, Tiwari V (2018). Strategies for combating bacterial biofilms: A focus on anti-biofilm agents and their mechanisms of action. Virulence.

[30] Giavarina D (2015). Understanding bland altman analysis. Biochem. Med..

[31] Myles, P. S. & Cui, J. Using the Bland–Altman method to measure agreement with repeated measures. 309–311 (Oxford University Press, 2007). 10.1093/bja/aem214 17702826

[32] Al-Momani H (2016). Microbiological profiles of sputum and gastric juice aspirates in cystic fibrosis patients. Sci. Rep..

[33] Al-Momani H (2017). Nontuberculous mycobacteria in gastrostomy fed patients with cystic fibrosis. Sci. Rep..

[34] Zeybel GL (2017). Ivacaftor and symptoms of extra-oesophageal reflux in patients with cystic fibrosis and G551D mutation. J. Cyst. Fibros..

[35] Rogers GB (2006). Use of 16S rRNA gene profiling by terminal restriction fragment length polymorphism analysis to compare bacterial communities in sputum and mouthwash samples from patients with cystic fibrosis. J. Clin. Microbiol..

[36] Héry-Arnaud G, Boutin S, Cuthbertson L, Elborn SJ, Tunney MM (2018). The lung and gut microbiome: What has to be taken into consideration for cystic fibrosis?. J. Cyst. Fibros..

[37] Rosen R (2015). 16S community profiling identifies proton pump inhibitor related differences in gastric, lung, and oropharyngeal microflora. J. Pediatr..

[38] Althuwaybi A (2021). A narrative review of the potential role of microaspiration and a dysregulated aerodigestive microbiome in lung disease. Ann. Esophagus..

[39] Huxley EJ, Viroslav J, Gray WR, Pierce AK (1978). Pharyngeal aspiration in normal adults and patients with depressed consciousness. Am. J. Med..

[40] Giannella R, Broitman S, Zamcheck N (1972). Gastric acid barrier to ingested microorganisms in man: Studies in vivo and in vitro. Gut.

[41] Rosina A (1982). Rapid anterograde movement of the fluorescent tracer fast blue: A new method for tracing central connections. Neurosci. Lett..

[42] Com G, Cetin N, O'Brien CE (2014). Complicated Clostridium difficile colitis in children with cystic fibrosis: Association with gastric acid suppression?. J. Cyst. Fibros..

[43] Littlewood JM, Wolfe SP, Conway SP (2006). Diagnosis and treatment of intestinal malabsorption in cystic fibrosis. Pediatr. Pulmonol..

[44] Proesmans M, De Boeck K (2003). Omeprazole, a proton pump inhibitor, improves residual steatorrhoea in cystic fibrosis patients treated with high dose pancreatic enzymes. Eur. J. Pediatr..

[45] van Horck M (2018). Risk factors for lung disease progression in children with cystic fibrosis. Eur. Respir. J..

[46] Al Momani H (2018). Risk factors for lung disease progression in children with cystic fibrosis. Eur. Respir. J..

[47] Rowbotham NJ, Smith S, Prayle AP, Robinson KA, Smyth AR (2018). Gaps in the evidence for treatment decisions in cystic fibrosis: A systematic review. Thorax.

[48] Reen FJ (2016). Bile signalling promotes chronic respiratory infections and antibiotic tolerance. Sci. Rep..

[49] Stewart PS, Costerton JW (2001). Antibiotic resistance of bacteria in biofilms. The Lancet.

[50] Yang L, Nilsson M, Gjermansen M, Givskov M, Tolker-Nielsen T (2009). Pyoverdine and PQS mediated subpopulation interactions involved in *Pseudomonas aeruginosa* biofilm formation. Mol. Microbiol..

[51] Snider DE (1985). Tuberculosis and gastrectomy. Chest.

[52] McDonnell MJ (2020). Current therapies for gastro-oesophageal reflux in the setting of chronic lung disease: State of the art review. ERJ Open Res..

