# Supplementary material for: Exposure to bile and gastric juice can impact the aerodigestive microbiome in people with cystic fibrosis
Source: Sci Rep. 2022 Jun 30;12:11114. doi: 10.1038/s41598-022-15375-4 (PMC9247099; doi:10.1038/s41598-022-15375-4)
Supplement: Supplementary file 1 — Supplementary Information. [file 41598_2022_15375_MOESM1_ESM.docx]

Supplementary table S1: The quality of assemblies assessment using QUAST (v4.4) and CheckM (v1.0.18)

| Patient | Source | GenomeID | Size (Mbp) | GC (%) | Completeness (%) | Contamination (%) | Contig number | Longest contig (bp) | N50 (bp) | N75 (bp) |
| --- | --- | --- | --- | --- | --- | --- | --- | --- | --- | --- |
| 1 |  | GM05 | 6673597 | 66.29 | 99.51 | 0.11 | 83 | 438886 | 139837 | 100279 |
| 1 |  | GM08 | 6666292 | 66.3 | 99.59 | 0.11 | 85 | 434060 | 139840 | 92229 |
| 1 |  | GM10 | 6649379 | 66.28 | 99.68 | 0.11 | 100 | 361983 | 136934 | 78679 |
| 12 |  | GM104 | 7025294 | 66.19 | 99.68 | 2.07 | 87 | 407313 | 149127 | 90617 |
| 12 |  | GM105 | 6906056 | 66.16 | 99.57 | 0.45 | 76 | 483407 | 192073 | 98465 |

**Appendix 1: Reflux Symptoms Index (RSI)**

| Within the last month, how did the following problems affect you?  0 = no problem 5= severe/frequent problem | | | | | | |
| --- | --- | --- | --- | --- | --- | --- |
|  | 0 | 1 | 2 | 3 | 4 | 5 |
| Hoarseness or problem with your voice |  |  |  |  |  |  |
| Clearing your throat |  |  |  |  |  |  |
| The feeling of something dripping down the back of your nose or throat |  |  |  |  |  |  |
| Retching or vomiting when you cough |  |  |  |  |  |  |
| cough or shortness of breath on first lying down or bending over |  |  |  |  |  |  |
| chest tightness or wheeze when coughing |  |  |  |  |  |  |
| heartburn, indigestion, stomach acid coming up (or medication =5) |  |  |  |  |  |  |
| A tickle in your throat, or a lump in your throat |  |  |  |  |  |  |
| cough or shortness of breath with eating (during or soon after) |  |  |  |  |  |  |
| cough or shortness of breath when you get out of bed in the morning |  |  |  |  |  |  |
| cough or shortness of breath brought on by singing or speaking (telephone) |  |  |  |  |  |  |
| coughing more when awake rather than asleep |  |  |  |  |  |  |
| a strange taste in your mouth |  |  |  |  |  |  |

**Appendix 2**

1. **Establishing effects of pepsin and acidity level on *Pa*.**

Following incubation, the *Pa* cultures were used to produce standardised suspensions of the microbes. They were suspended in 2ml PBS, and a densitometer was used to generate a 0.5 McFarland standard comparable density suspension (1.5 *10⁸ cfu/ml).

A 10µl *Pa* Inoculum was pre-incubated with 1 ml solution containing the test substances, with 20 µl being taken at durations of 0 minutes, 5, 30, 60 and 120 minutes. These underwent dilution in 1.98ml PBS, pH 7.4. 50 µl of the neutralised suspension of bacteria was taken for plating in blood agar, and this was done in triplicate, to give about 50-60 *Pa* colonies. Viable counts were determined after culturing at 37°C for 24 h under aerobic conditions.

**B. Biofilm assay: microtiter-plate test**

For the biofilm assay, cultures of the four *Pa* strains were used to produce standardised suspensions. This was done using Tryptone Soy Broth (TSB), using 250µl aliquots for each suspension, to which was added 20 µl aliquots of the bacteria in suspension with TSB and bile acids, leaving 270µl in each well in the microtiter tray. Each test was performed in triplicate. In addition, positive and negative control wells were used, with positives containing *Pa* and TSB with the omission of bile salts, and negatives containing TSB only.

This preparation was followed by aerobic incubation at 37°C for 24 hours, before aspirating the wells and washing each in sterile physiological saline (250µl) 3 times. Vigorous shaking of the plates was carried out for the purpose of removing any bacteria which had not adhered. Those microbes which were left underwent fixing using 99% methanol, at 200 µl for each of the wells. The plates were left for 15 minutes, before emptying them and letting them dry. Five minutes’ staining was then carried out for each plate, using 2% Hucker crystal violet, suitable for Gram-stain use, at 200 µl for each of the wells. Excess staining material was removed through rinsing plates with flowing water from the tap. The plates were then allowed to dry once more, and resolubilisation of the cell-bound dye was done using 33% (v/v) glacial acetic acid, at 160 µl for each of the wells.

Optical density (OD) measurement was carried out for all the wells via an automated reader, the ICN Flow Titertek Multiscan Plus. Readings were taken at three stages: firstly, prior to incubating the samples (OD 600nm); secondly, post-incubation for growth assessment (OD 600 nm); and finally, once the biofilm assay had been completed (OD 570nm). The ratio selected was 570/600, for normalisation of the measure of biofilm formed against growth of bacteria.


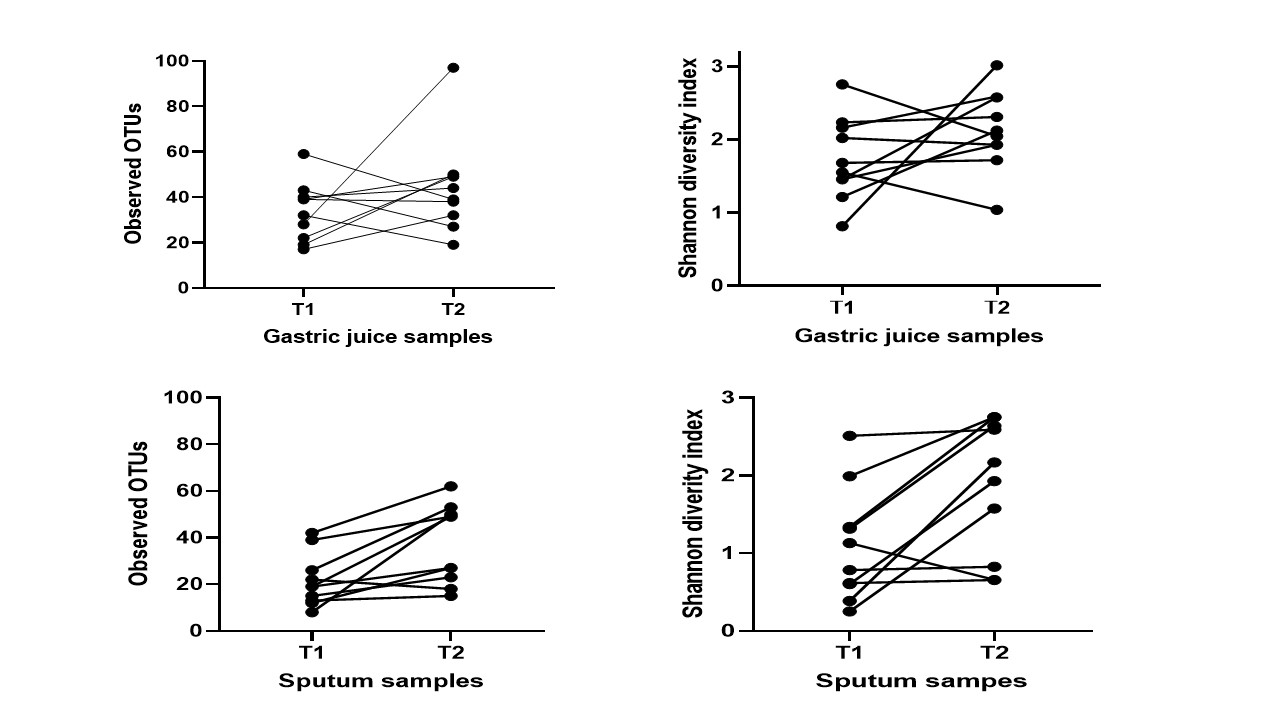


Figure S1: Paired data plot showing the individual observed OTUs and Shannon Diversity values of each microbiome at T1 and T2 with lines indicating how each point relates in time.

Figure S2: PERMANOVA analyses comparing bacterial profiles in CF gastric juice and sputa which indicates that the centroids and spread of the profiles overlapped between the groups (P=0.08).


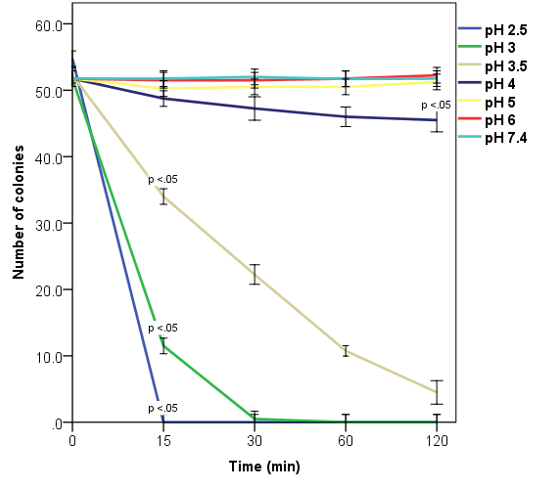


Figure S3: Effect of different pH levels (2.5-7.4) on subsequent growth of the 4 strains P. aeruginosa (S27, S33, S34 and Pa14) measured by average number of colonies for the 4 strains (X-axis) after incubation period range from 15 to 120 minutes (Y-axis). Number of Pa colonies at pH at 7.4 were used for comparison (control).


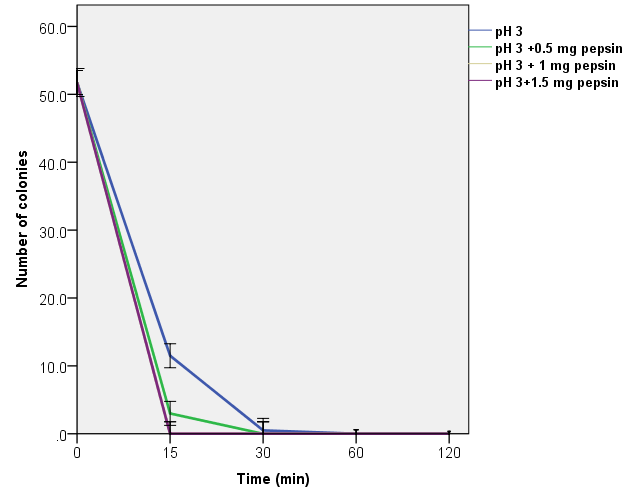


Figure S4: Effect of pH and pepsin (0.5-1.5 mg/ml) on *PA* viability as indicated by colony counts. There was a significant drop (F (2, 9) = 7.29, p <. 02) in the number of colonies when pepsin (at concentrations of 0.5-1.5mg ml^-1^) added to PBS with pH 3 (T-value = 27.71, P-value = 0.000, DF = 6).


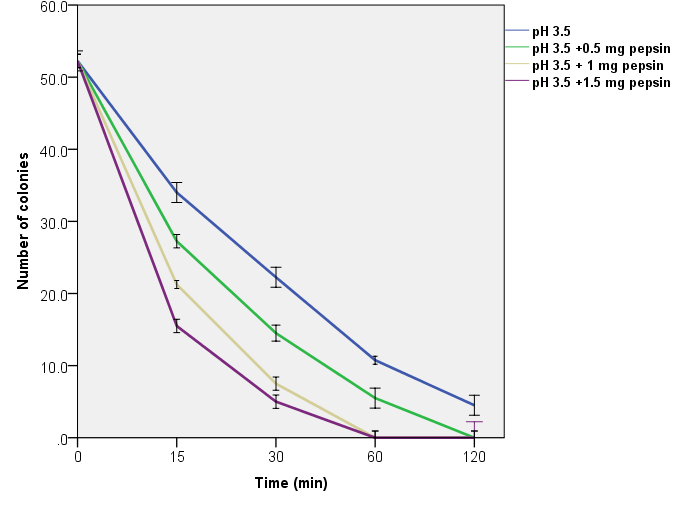


Figure S5: Effect of pH 3.5 and pepsin (0.5-1.5 mg/ml) on *PA* viability as indicated by colony counts. There was a statistically significant drop in the number of colonies when pepsin was added to the PBS at all pepsin concentrations used (except at 120 minutes at 0.5 mg/ml) using ANOVA test with post hoc (F (3, 11) = [21.40], P<0.0001, P-values been corrected for multiple hypothesis testing using Bonferroni correction method). At 15 minutes, there was a statistically significant drop in the number of colonies at all pepsin concentrations compared to the samples without pepsin (0.5 mg: T-value = 11.55, P-value = 0.007, DF = 2), 1mg: T-value = 21.89, P-value = 0.002, DF = 2) and 1.5mg: T-value = 31.88, P-value = 0.001, DF = 2). At 30 minutes, there were statistically significant drops in colony counts at all pepsin concentrations compared to the number of colonies without pepsin (0.5mg: T-value = 13.01, P-value = 0.006, DF = 2). 1 mg: T-value = 43.96, P-value = 0.000, DF = 3) and1.5 mg: T-value = 28.70, P-value = 0.001, DF = 2). At 60 minutes, there was statistically significant decrease in the number of colonies at pepsin concentration 0.5 mg (T-value = 41.05, P-value = 0.001, DF = 2). There was no statistically significant difference in colony counts with 0.5mg/ml pepsin compared to the number of colonies without pepsin at 120 minutes.


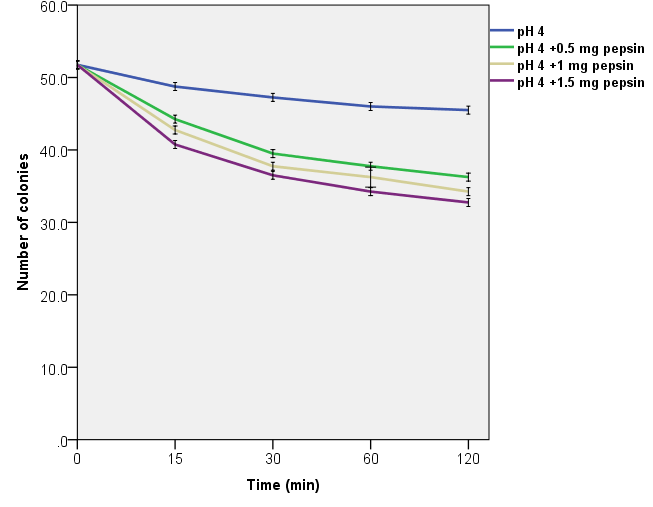


Figure S6: Effect of pH 4 and pepsin (0.5-1.5 mg/ml) on *PA* viability, as indicated by colony counts. There was a statistically significant drop in the number of colonies when pepsin was added to the PBS at all concentrations and at all time points. At 15 minutes, there was a significant decrease in the number of colonies when was pepsin added at all concentrations compared to incubating the *PA* without pepsin (F (4, 12) = [25.60], P<0.0001, P-values been corrected for multiple hypothesis testing using Bonferroni correction method) (0.5mg/ml: T-value = 10.28, P-value = 0.009, DF = 2). 1mg: T-value = 14.01, P-value = 0.005, DF = 2, at 1.5mg: T-value = 18.43 P-value = 0.003, DF = 2). At 30 minutes (0.5mg: T-value = 25.09, P-value = 0.002, DF = 2, at 1mg/ml :T-value = 58.18, P-value = 0.000, DF = 4, at 1.5mg: T-value = 34.74 P-value = 0.001, DF = 2). At 60 minutes (0.5mg: T-value = 13.93, P-value = 0.005, DF = 2). 1mg/ml: T-value = 16.09, P-value = 0.004, DF = 2, at 1.5 mg/ml (T-value = 19.41, P-value = 0.003, DF = 2). At 120 minutes (0.5mg/ml: T-value = 27.33, P-value = 0.000, DF = 3), at 1mg/ml: T-value = 33.27, P-Value = 0.000, DF = 3). At 1.5 mg/ml: T-value = 40.85, P-value = 0.001, DF = 2).


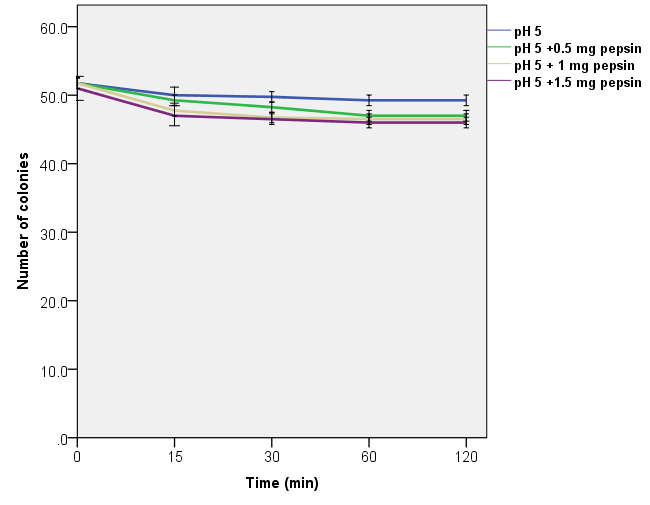


Figure S7: Effect of pH 5 and pepsin (0.5-1.5 mg/ml) on *PA* viability as indicated by colony counts. There was no statistically significant drop in colony counts when pepsin was added to the PBS at any concentration used (F (3,16) = [1.906], P=0.169).
